# New trends in surgery for colorectal liver metastasis

**DOI:** 10.1002/ags3.12810

**Published:** 2024-04-26

**Authors:** Philipp Kron, Peter Lodge

**Affiliations:** ^1^ Department for General and Transplantation Surgery University Hospital Tuebingen Tuebingen Germany; ^2^ St. James's University Hospital, Leeds Teaching Hospitals NHS Trust Leeds UK

**Keywords:** hepatectomy, liver, liver transplantation, metastasis, neoadjuvant therapy

## Abstract

By presenting the most up‐to‐date findings and incorporating the latest evidence, this article seeks to present a comprehensive guide for navigating the complexities inherent in the management of colorectal liver metastasis. It aims to serve as a valuable resource offering clinicians and healthcare professionals an understanding of the diverse modalities and approaches available for treating this challenging and multifaceted disease. In an era of rapidly evolving medical knowledge, this article examines the latest insights to make informed decisions in the realm of colorectal liver metastasis management. The article does not only highlight the up‐to‐date knowledge but also provides the evidence for existing therapeutic strategies. This practical tool provides evidence‐based recommendations to clinicians, thereby contributing to the ongoing advancement of effective treatment strategies for this challenging disease.

## Graphical abstract

In an era of rapidly evolving medical knowledge, this article examines the latest insights to make informed decisions in the realm of colorectal liver metastasis management. The article not only highlights the up‐to‐date knowledge but also provides the evidence for existing therapeutic strategies.

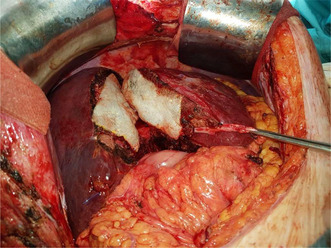

## 1 INTRODUCTION

Colorectal cancer is the third most common cancer worldwide, with over 1 million new cases per year.^1^ Death is often caused by colorectal liver metastases (CRLM), which affect up to 50% of patients, yet few with metastatic disease are amenable to liver resection even today.^2^, ^3^ Multimodal treatment combining surgical and nonsurgical strategies offer the best chance for cure in CRLM. Treatment approaches for each patient should be discussed within a multidisciplinary team (MDT) to define a personalized treatment strategy.^4^

It is accepted that resection for CRLM is both efficient and cost‐effective, improves survival, and has provided acceptable quality of life.^5^, ^6^ In recent years, the shift has been away from dealing with limited disease and obtaining wide margins of clearance and more towards ensuring complete excision with preservation of an adequate future liver remnant (FLR). Thus, the FLR has become more of a priority than the volume or distribution of the disease. This review outlines the up‐to‐date treatment strategies for colorectal liver metastases, many of which challenge accepted dogma.

## 2 MDT DECISION MAKING

The liver cancer MDT should play a pivotal role in determining therapeutic strategies for CRLM. Evidence is lacking that MDT discussions have significantly contributed to superior survival, but MDTs provide an essential platform for reviewing these cases and making therapeutic plans as new treatment strategies develop, providing education for MDT members and individualizing treatment according to patient fitness and distribution of disease.^5^, ^6^, ^7^ As a result of the increasing complexity of approaches, treatment pathways in CRLM remain inhomogeneous, even among academic centers; international high‐level consensus statements and widely accepted guidelines are needed. In the interim, it remains essential that all cases of CRLM are deliberated within MDTs specializing in their treatment. This approach continues to be the most effective method thus far.^7^ To provide a holistic approach, the core MDT should ideally include the following specialties: medical oncology, pathology, radiology, radiation oncology, colorectal and hepato‐pancreato‐biliary (HPB) surgery, and gastroenterology, but the reality is that a smaller group can make appropriate decisions.^8^ The MDT not only plays a key role during the initial assessment of resectability, but it also has an ongoing task of reassessment in CRLM, especially in patients being initially defined as unresectable.

### 2.1 Chemotherapy

There is little doubt that resectable CRLM in patients with favorable biology should be treated by upfront liver resection. The role of neoadjuvant chemotherapy is more difficult to define.

Evidence from the European Organization for Research and Treatment of Cancer (EORTC) trial 40 983 supports the hypothesis that perioperative chemotherapy prolongs recurrence‐free survival (RFS), but no studies have demonstrated a superior overall survival when applying neoadjuvant chemotherapy for CRLM.^9^, ^10^, ^11^ Furthermore, a subset of primarily unresectable tumors can be converted to resectability by systemic therapy. The benefit of this approach has been demonstrated in several retrospective studies.^12^, ^13^, ^14^ The majority of patients with colorectal metastases are unresectable at first presentation.^15^ In the past they have been treated in a palliative setting. There is evidence reporting the use of “neoadjuvant”/induction chemotherapy and the effectiveness of downsizing CRLM with resection rates up to 30% and 5y survival rates of up to 50%.^16^, ^17^, ^18^ Induction therapy has the aim of converting an initial unresectable disease into a resectable status. Resectable is defined by complete removal of the disease as well as leaving an adequate FLR.

**FIGURE 1:**
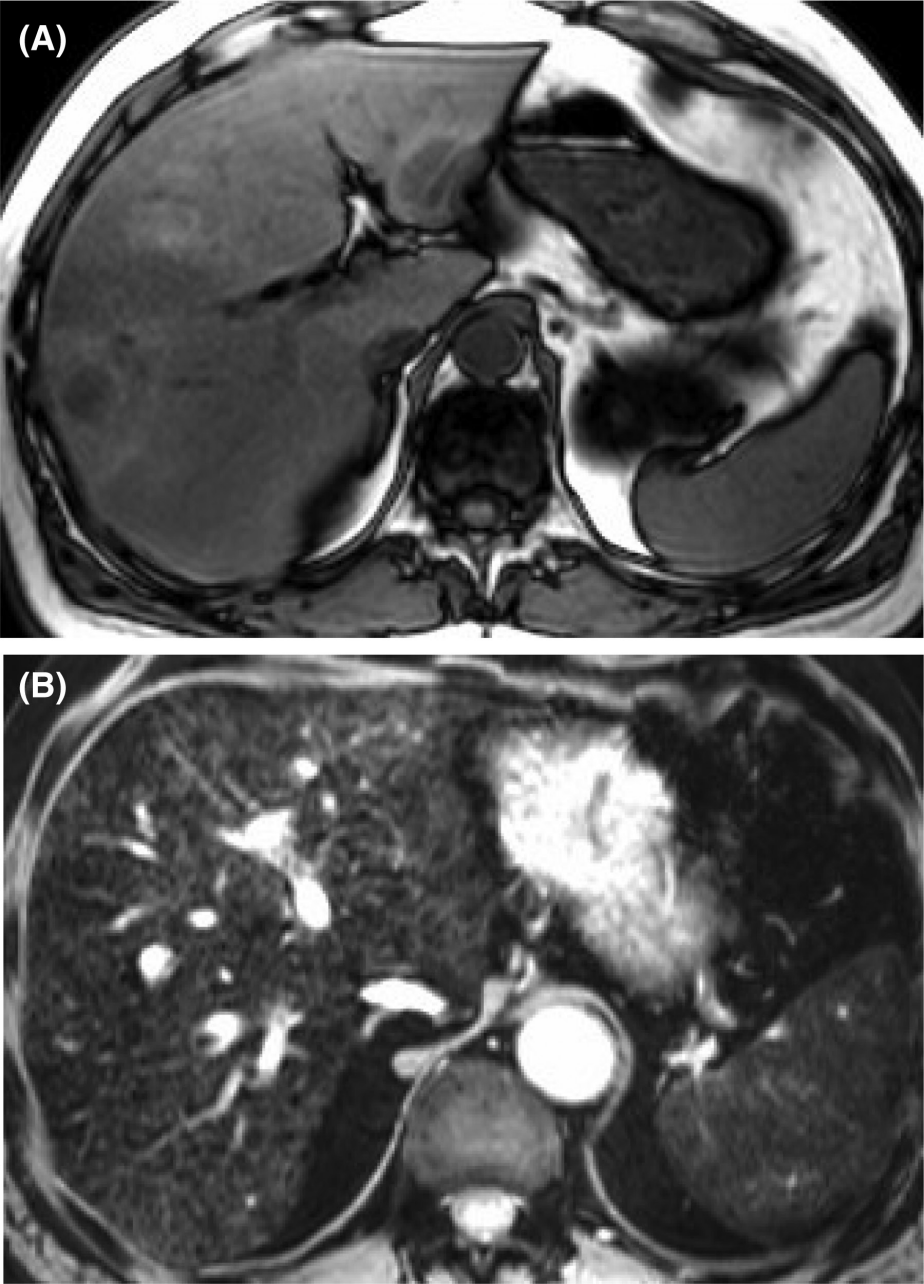
Liver MRI shows steatosis following irinotecan‐based chemotherapy (A) and sinusoidal obstructive syndrome (SOS) after oxaliplatin (B).

As surgeons we need to be aware of the potential negative impacts of neoadjuvant chemotherapy on liver resection outcomes. The negative correlation between postoperative infective complications and long‐term outcome is established.^19^ Irinotecan‐based chemotherapy is associated with steatosis and steatohepatitis, and fatty liver disease is associated with a higher risk for CRLM recurrence, but the ultimate impact of chemotherapy in this regard is unknown (Figure 1).^20^ Steatohepatitis has a significant impact on postoperative outcomes following liver surgery. The MD Anderson group have shown that patients suffering from irinotecan‐induced steatohepatitis had a significantly higher 90‐d mortality following hepatic surgery for colorectal liver metastases compared to patients without steatohepatitis.^21^ Oxaliplatin is associated with sinusoidal obstructive syndrome (SOS), which can present operative challenges for bleeding related to intrahepatic venous hypertension, and this can progress to portal hypertension in some patients, further increasing perioperative risk (Figure 1).

In fact, two recent randomized controlled trials that showed improvement in progression‐free or disease‐free survival have failed to provide evidence for an improvement in overall survival. The results of the JCOG0603 trial have signaled the end of an era in which perioperative or postoperative chemotherapy can be considered the default standard for patients with resectable CRLM,^14^, ^22^ but there is little current evidence that the views of medical oncologists are changing in this regard, so MDT discussion for individual patients remains important.

## 3 PREOPERATIVE WORKUP

Preinterventional imaging in CRLM is of utmost importance to define the most effective treatment regimen for each individual patient. The standard investigation worldwide independent of primary or recurrent disease in CRLM is a contrast‐enhanced computed tomography (CT) scan. The role of liver contrast‐enhanced magnetic resonance imaging (MRI) and the gain of additional information influencing the treatment algorithm was unclear until relatively recently. In a multicentric study the effect of liver contrast‐enhanced MRI and the effects on the initial treatment plan were assessed.^23^ In their conducted trial among 14 centers, the primary outcome measure, change in local treatment plan on the basis of the contrast‐enhanced MRI, was observed in 92 out of 298 participants (31%).^23^ A central blinded expert panel evaluated 297 cases and saw an indication for a change of treatment plan in 34% of the included patients. In detail, among the centers the modification of the initial treatment plan varied 14%–57%. In 13%, more extensive local therapy was needed, 4% needed less extensive local therapy, in 11% the decision for curative intention was revoked, including 3% of 298 patients in which suspected CRLM were found to be benign on MRI. In 5% that were initially amenable for local treatment, induction chemotherapy was conducted after the MRI results were analyzed. There was no subgroup of patients in which contrast‐enhanced MRI had no clinical effect on the staging. Therefore, based on the available evidence, routine workup in all patients with CRLM should be performed via contrast‐enhanced MRI to assess and adapt the treatment plan accordingly. The role of positron emission tomography (PET)‐CT continues to be debated.

## 4 DISAPPEARING COLORECTAL LIVER METASTASIS

There is currently no clear recommendation on whether the “disappearing” CRLM should be resected or left in situ. The major question relates to defining this as a complete radiological or pathological response. The treatment of this special entity is complex and requires a tailor‐made approach. Features such as patient characteristics, the feasibility of technical R0 resection of the original site of the metastasis, and other tumor characteristics (eg, tumor burden) are essential to consider.^24^ So far, there is only limited evidence assessing the management and follow‐up of disappearing CRLM. There is existing evidence showing that a high percentage of disappearing CRLM are detected during surgery (64%) and that a high percentage of the liver metastases that were resected, contained viable tumor cells.^25^ A study from 2016 comparing resection and conservative treatment with follow‐up scans showed a longer recurrence free survival in the resection group (483 d vs 360 d; ns).^26^ This corroborates previous work assessing surgery versus watchful waiting, which did not show a difference in overall survival, but did demonstrate an advantage in intrahepatic recurrence after 3 y in the resection group.^27^ Up‐to‐date evidence suggests that resection of all sites of disease has an advantage in terms of intrahepatic recurrence.^28^

In conclusion, the management of disappearing CRLM remains a matter of debate. Due to the dilemma that not all metastases are revealed in preoperative scans, and previous sites may still harbor viable cancer cells, a complex challenge arises. In an ideal world, all sites / previous sites of disease should be resected. Even in the era of parenchymal‐sparing resection this may lead to a significant parenchymal loss, not leaving an adequate FLR. Therefore, a tailor‐made approach is necessary and further research and evidence is needed to answer this question eventually. Until then, it seems reasonable to resect all areas of concern, if feasible.

## 5 SURGICAL DECISION MAKING

**FIGURE 2:**
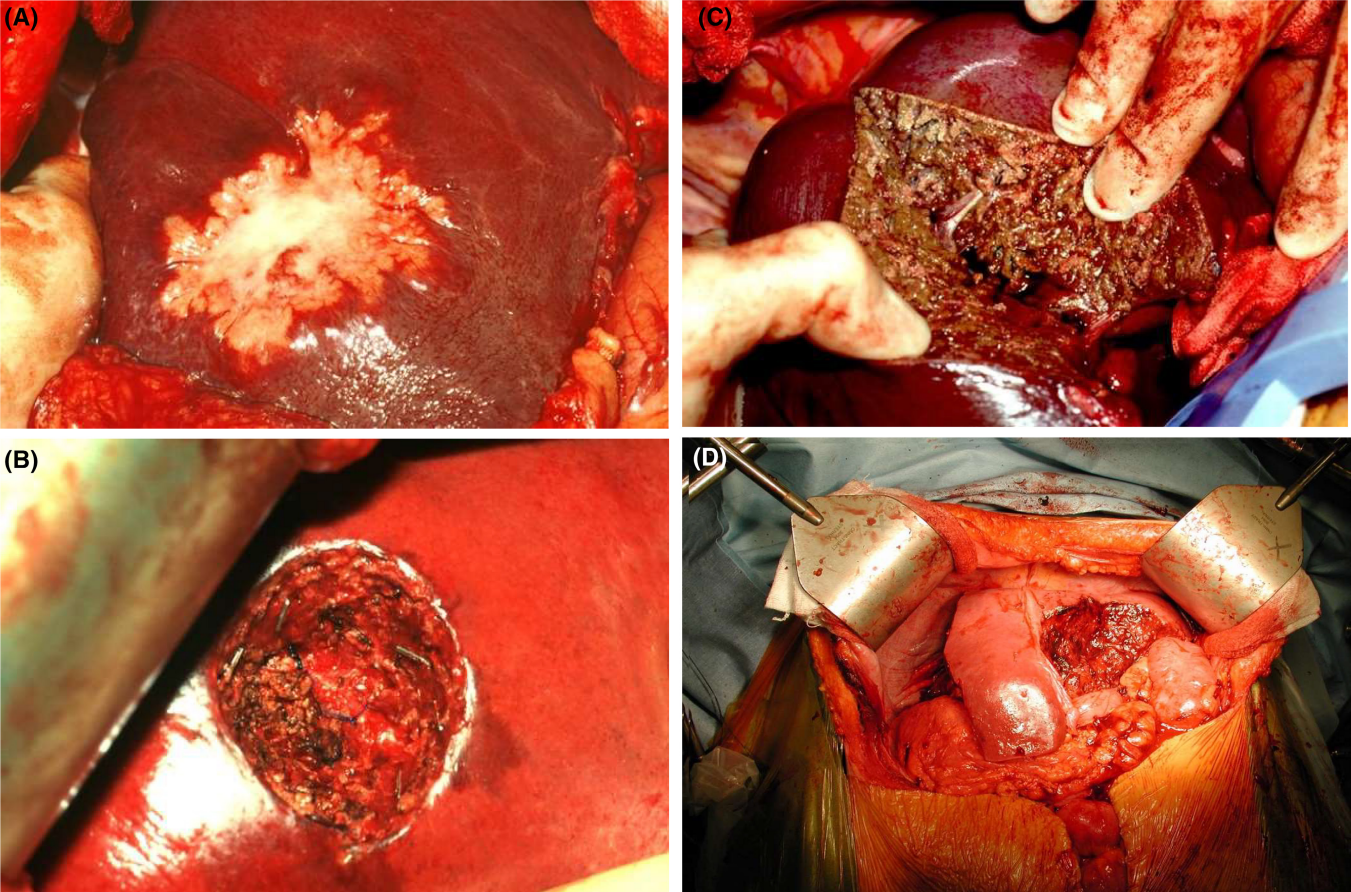
Surgical options for CRLM (A) include parenchymal‐sparing nonanatomic metastasectomy (B), anatomic resection (C), or a combined approach for multiple CRLM (D).

Resection for CRLM is no longer limited by the number nor the location of the metastases, and it is becoming accepted that limited extrahepatic disease, often called oligometastatic, should not be a barrier to surgery. Therapeutic decision making is based on two principles: first, achieving R0 resection, and second, leaving a sufficient FLR to ensure postoperative survival^29^, ^30^ (Figure 2). The size of the FLR is dependent on the state of the liver, varying between 20% and 40% from a healthy to a postchemotherapy liver.^16^, ^17^, ^18^

The first consideration is whether to carry out a parenchymal‐sparing nonanatomic resection or a more classical anatomic approach such as a hemi‐hepatectomy or extended hemi‐hepatectomy (Figure 2). The parenchymal‐sparing approach is associated with less perioperative risk and seems adequate as long as clear margins can be achieved. However, patients undergoing such surgery more often require re‐resection for occult micro‐metastatic CRLM that may have been included in a more major resection initially.^19^ On the other hand, a more major resection initially may deny the patient a future resection if disease recurs in the FLR, due to anatomic considerations. Thus, there is a balance to be achieved in resection strategy decision making.

The reality is that in order to control CRLM, a combination of techniques is often employed, and outcomes for major anatomic resection with added parenchymal‐sparing techniques can be similar. Complex vascular resection and reconstruction is rarely indicated for CRLM, but is justifiable if it enables complete resection^31^, ^32^ The use of ischemia techniques such as the Pringle maneuver and total vascular exclusion have enabled these developments and it is clear that when used appropriately there is no detriment to long‐term survival.^32^

**FIGURE 3:**
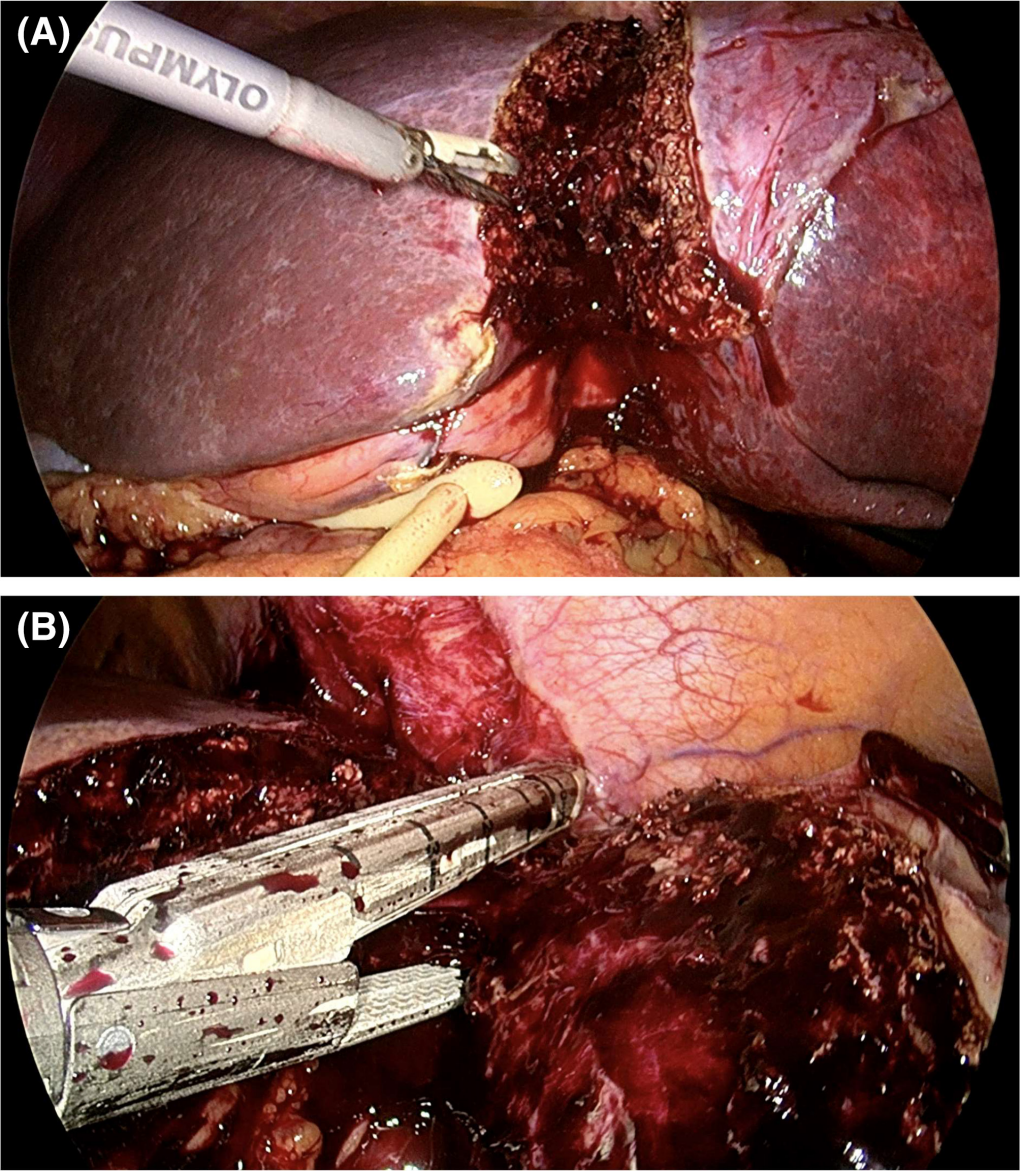
Laparoscopic liver resection. In this example, a Foley catheter encircles the portal hilum in case a Pringle maneuver was needed (portal inflow constriction) and a Thunderbeat combined ultrasonic and advanced bipolar energy technology (Olympus, Japan) was used for parenchymal transection (A), with an endo‐GIAsurgical stapler (Medtronic, USA) used for the final stages to secure major vasculature (B).

Laparoscopic and robotic surgery approaches to liver resection are now well developed, with similar long‐term outcomes and reduced perioperative morbidity (Figure 3).^33^, ^34^, ^35^

**FIGURE 4:**
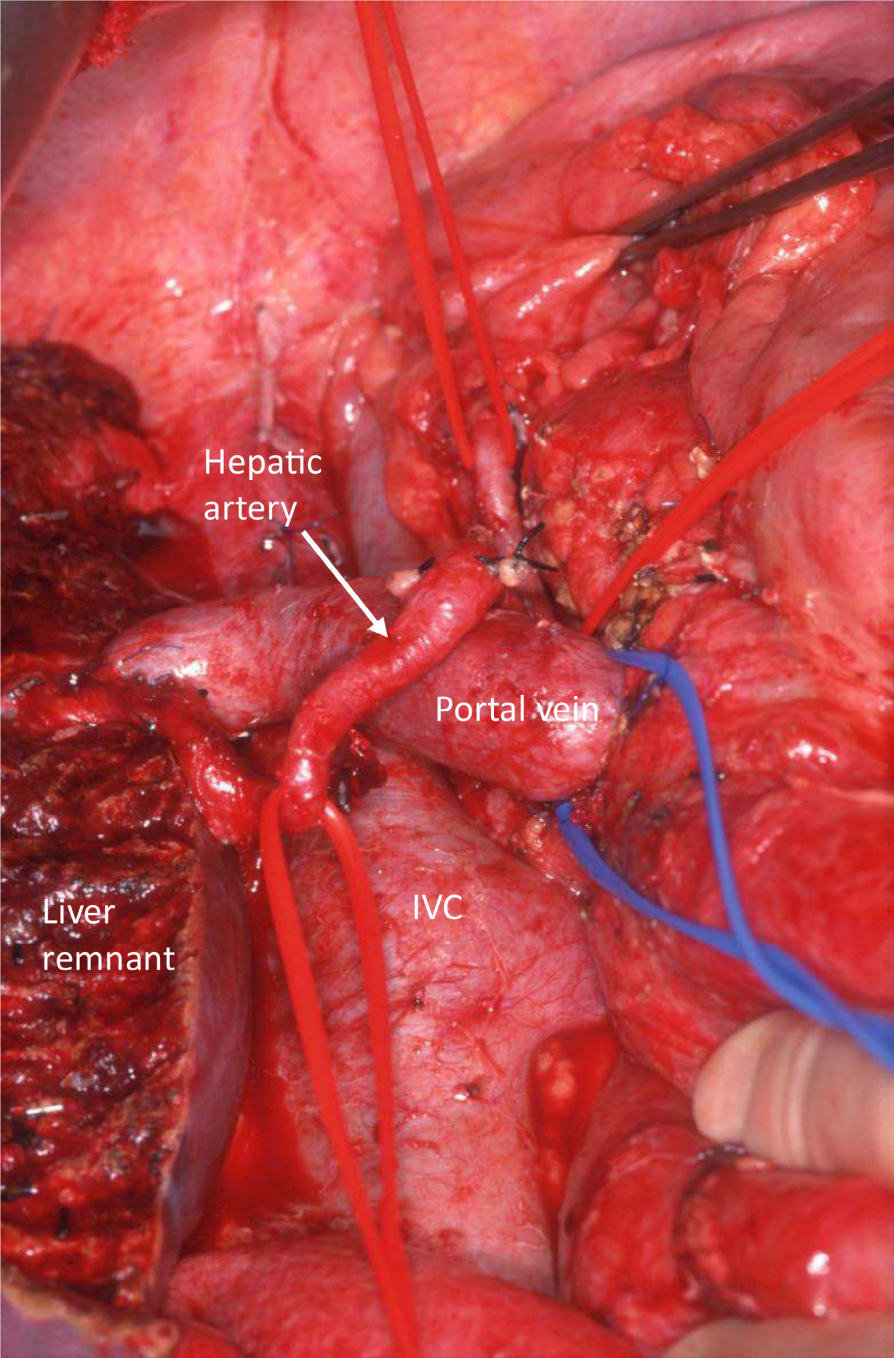
Regional lymphadenectomy combined with liver resection. Then portal vein and hepatic artery were skeletonized in this example of left hemihepatectomy, which also includes bile duct resection. The duodenum and head of pancreas were retracted medially to allow a more extensive regional lymphadenectomy in this case. The degree of lymph node invasion in this advanced case meant that a more extensive lymph node dissection than usual was carried out, with resection of the left hepatic artery and gastroduodenal artery and the extra hepatic biliary tree. The reality for most cases is a less radical approach, with removal of regional lymph nodes, but no other structures.

Repeat hepatic resection is also justified, as is resection in the presence of oligometastatic disease.^36^, ^37^ Regional lymphadenectomy also has a place, particularly as lymph node involvement is easy to overestimate.^38^, ^39^ Sometimes, this may involve combined resection of the extrahepatic biliary tree (Figure 4), although this is not usually needed. Combined resection of extrahepatic bile ducts in the setting of insufficient FLR increases the incidence of postoperative severe complications. In addition, it can make it more difficult to perform another hepatic resection at the time of intrahepatic recurrence. Lymph node‐positive primary colorectal cancer and the problem of local recurrence remains a significant barrier to optimal CRLM resection long‐term results, so careful monitoring after surgery is of paramount importance so that re‐resection can be considered. Routine lymphadenectomy will be continued to be debated, as there is yet no clear benefit in terms of survival, whether through a direct effect of tumor clearance or through adjusting management if used as a prognostic marker.^38^ Our own practice is to just remove any suspicious nodes. We feel that combined hepatectomy and hepatic pedicle lymphadenopathy for CRLM in the setting of radiologically or clinically suspicious or enlarged lymph nodes is justified on the basis of our own findings that half of all suspected hepatic pedicle disease is benign on histology. There is no doubt that those with hepatic pedicle lymph node metastases do poorly when compared to those without extrahepatic disease; however, detecting these patients preoperatively is difficult. CT and MRI detect less than half of histologically proven metastases; however, PET‐CT possibly has a high positive predictive value, although the numbers in our own studies have been too small to make any further conclusions. There is no current role for routine lymphadenectomy; however, this is likely to change in the future where identifying patients with hepatic pedicle metastases can direct them towards aggressive therapy at an earlier stage.

## 6 THE FUTURE LIVER REMNANT (FLR)

**FIGURE 5:**
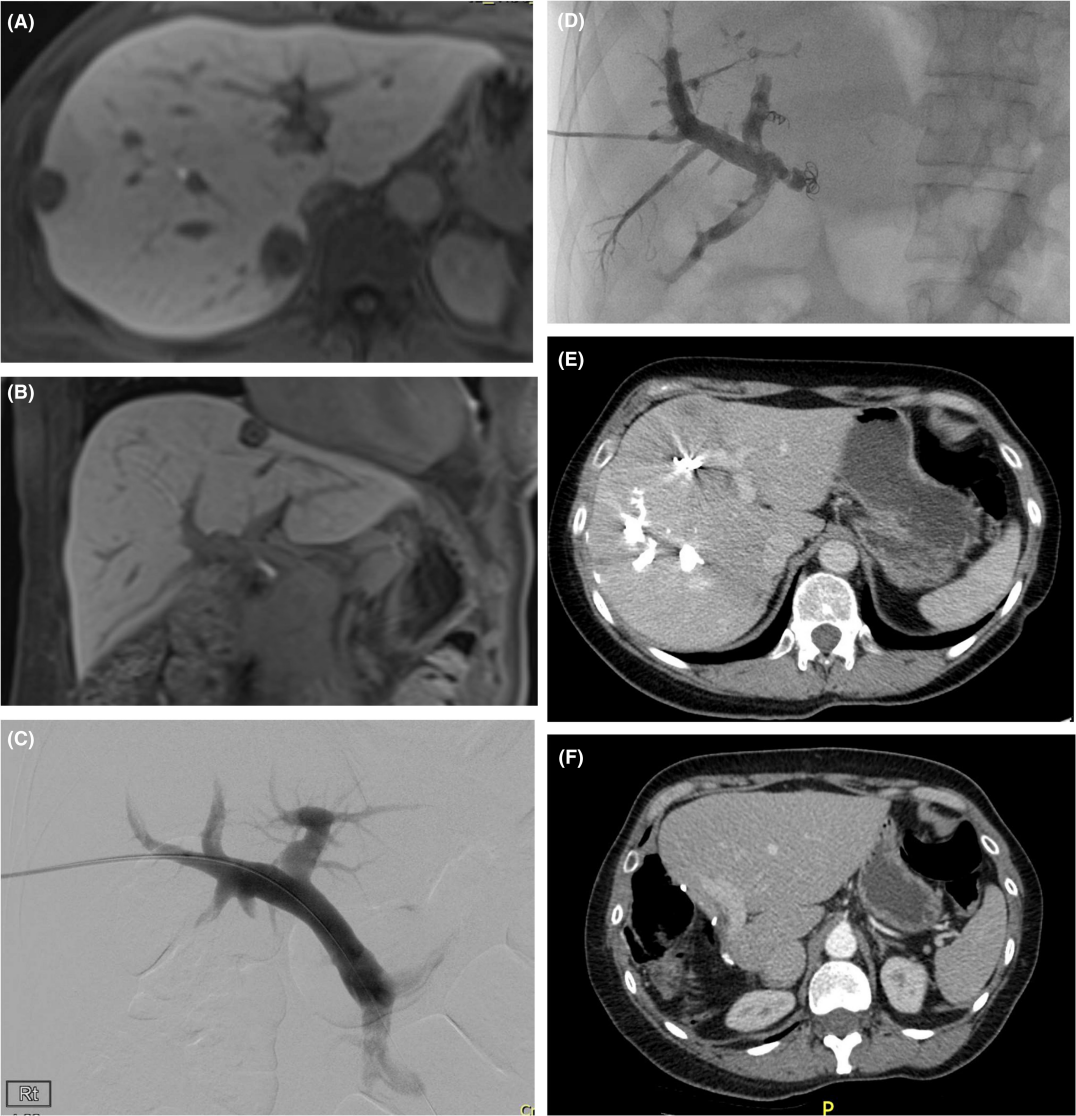
MRI showing multiple CRLM (A,B) requiring right hepatic trisectionectomy (resection of segments 4–8). PVE (C,D) resulted in FLR hypertrophy (E), allowing successful resection and further hypertrophy (F).

One significant challenge for patients with extensive CRLM is ensuring the FLR is adequate. This is an important consideration, as a poor decision can lead to significant postoperative morbidity and a risk of mortality due to liver failure (Figure 5). CT volumetry offers a degree of reassurance, but better functional tests for FLR are needed.

Portal vein embolization (PVE), is a useful approach to ensuring an adequate FLR (Figure 5).^40^, ^41^ The principle is based on the fact that the liver has two blood supplies: in simple terms, the hepatic artery provides oxygen and the portal vein nutrients; so embolization of the right portal vein before right hemi‐hepatectomy, for example, will cause right liver atrophy and left liver hypertrophy over the course of about 4 weeks.^40^, ^41^ If right hepatic trisectionectomy (right hemihepatectomy extended to include resection of segment 4) is planned, then the PVE should also include segment 4.^42^ Rates of hypertrophy are affected by age, underlying liver parenchymal disease, and chemotherapy.

PVE is increasingly combined with hepatic vein embolization (HVE). Evidence comparing HVE and PVE versus PVE alone could show a significantly higher volume in FLR as well as a significant higher kinetic growth rate compared to the PVE alone group.^43^ The recent DRAGON trial involved 39 patients who had undergone PVE/HVE and 160 had PVE alone. The PVE/HVE group had better hypertrophy than the PVE group (59% vs 48% respectively; *P =* 0.020) and resectability (90% vs 68%; *P =* 0.007). Major complications (26% vs 34%; *P =* 0.550) and 90‐d mortality (3% vs 16% respectively, *P =* 0.065) were comparable. Multivariable analysis confirmed that these effects were independent of confounders, so in this study PVE/HVE achieved better FLR hypertrophy and resectability than PVE alone.^44^ Further studies have been able to further confirm these results, related to a significantly higher kinetic growth rate.^44^, ^45^, ^46^ To have a uniform mode of reporting and a more certain comparability among conducted studies it was suggested to report the kinetic growth rate in the future, because this parameter is also a predictive value for posthepatectomy liver failure, giving us additional functional information among different treatment modalities.^47^, ^48^

## 7 MULTISTAGE SURGICAL APPROACHES

Sometimes, a two‐stage surgical strategy is appropriate for CRLM, and there is no doubt that this concept has extended the bounds of liver resection. Classical two‐stage hepatectomy combines an initial resection with contralateral portal vein embolization or ligation, followed by a second resection 1–2 mo later. Portal vein ligation appears to cause a similar regenerative FLR effect.^49^ There is an inevitable dropout rate between stages due to tumor progression, but in more than 75% of cases a successful outcome can be achieved, and long‐term results appear to justify this approach.^50^

**FIGURE 6:**
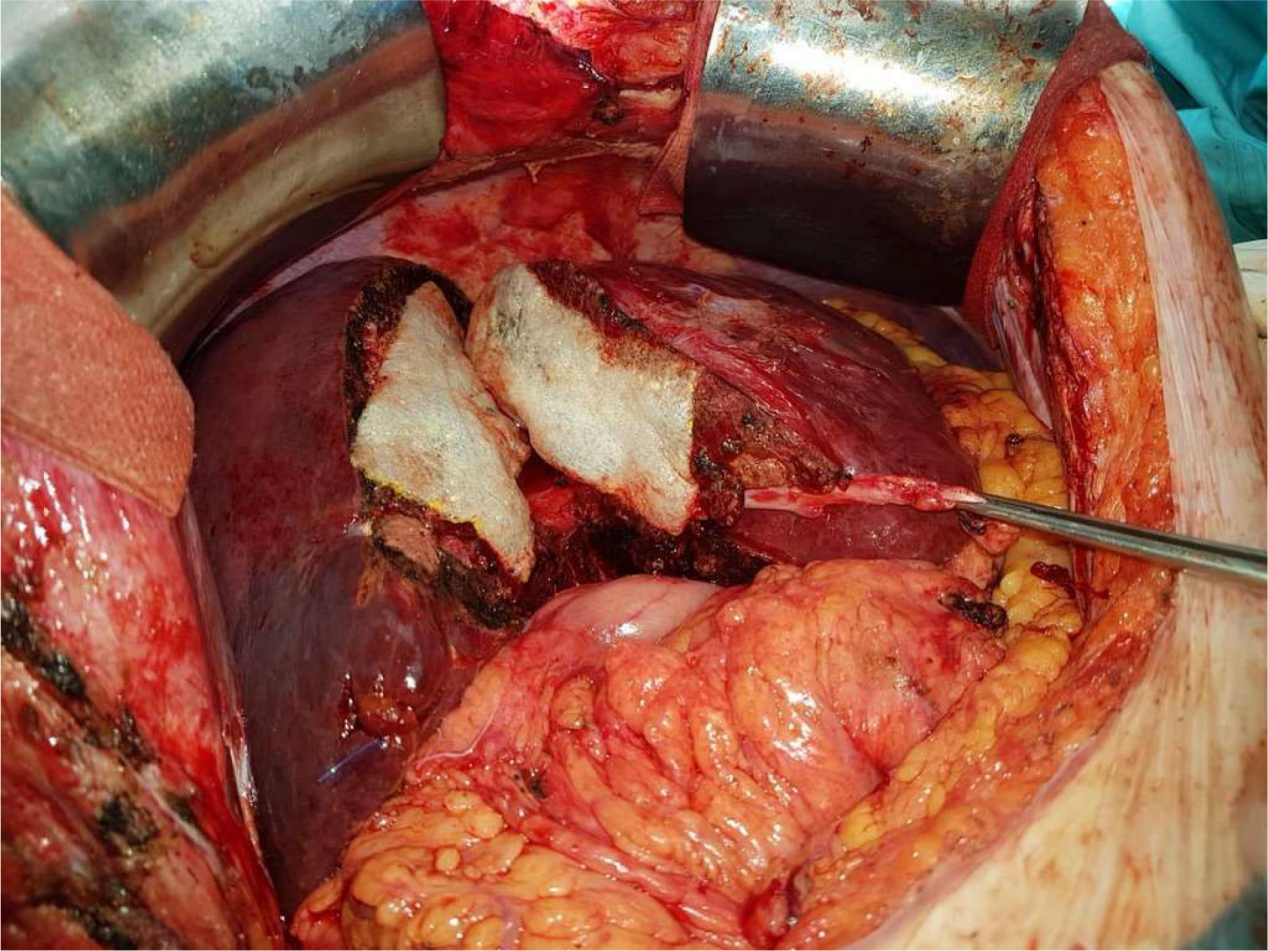
Completion of stage 1 ALPPS: the use of hemostats such as TachoSil (Takeda Pharmaceuticals, Japan) between the cut surfaces prevents adhesion before the second stage. Features of postchemotherapy liver injury are apparent.

A more recently described approach is known as ALPPS: associating liver partition and portal vein ligation for staged hepatectomy (Figure 6).^51^ The principle is to use both portal vein ligation and liver parenchymal transection to engender a more rapid FLR hypertrophy, enabling the second‐stage surgery to be carried out with a lesser time interval between the stages, with most authors describing this as 7–10 d. However, although associated with a high rate of progression to second stage and a higher complete resection (R0) rate, this procedure is associated with higher perioperative risk.^52^ Preliminary data indicate that mortality rates ranged from 9% to 12%, with major complications (≥3 Clavien–Dindo) reported at 27%. These figures surpass the mortality rates observed in traditional two‐stage hepatectomy procedures.^51^, ^53^, ^54^ The higher risk for ALPPS has led to several modifications, with improved perioperative morbidity and mortality, and longer‐term results are now acceptable.^52^ Further studies from the ALPPS registry could show a significant decrease in perioperative mortality to 4%.^55^ A recent meta‐analysis did not show any significant differences in 90‐d mortality rates or major morbidity between ALPPS and other modalities, including two RCTs.^56^, ^57^, ^58^

The ALPPS registry has enabled a large experience to be analyzed and this has shown that patient selection is paramount for good outcomes.^59^ In our experience, safety can be improved by a slight delay in the interstage interval; we have had no deaths and minimal mortality with a 14‐d interval between stages: d‐ALPPS.^60^ The initial experiences of ALPPS had a high morbidity and mortality that could have been decreased by meticulous patient selection, and delay of the second step if necessary.^61^ Therefore, in a single‐center cohort study we delayed the second stage to at least 14 d (delayed ALPPS) and compared this approach to conventional two‐stage hepatectomy. All patients in the cohort suffered from CRLM. We showed a significantly higher R0 resection rate in the d‐ALPPS group. In our cohort we had zero mortality in the d‐ALPPS group (or the conventional two‐stage hepatectomy group). We think by delaying the interstage interval mortality can be eliminated by decreasing the risk of posthepatectomy liver failure without compromising oncological outcomes, demonstrated by the significantly lower R1 resection rates compared to conventional two‐stage hepatectomy. Furthermore, we showed a tendency to higher completion rates in the ALPPS group, although this did not reach statistical significance.

In conclusion, we think that delaying the interstage interval to 14 d or more in ALPPS is safe in terms of morbidity and mortality. We postulate that the improved early outcomes in terms of reduced posthepatectomy liver failure are related to allowing more time for not only hypertrophy, but improved functionality in the FLR before the final resection, but other factors related to recovery from the first stage resection may also play a part. The reality is that we don't know, but what is clear is that this delay does not affect the completion rate or “oncosurgical” outcomes. Therefore, this technique might be ideal for new centers implementing ALPPS or centers with no access to functional liver tests such as LiMAx.

Doubters remain for ALPPS but the recent LIGRO Trial has compared this technique with classical two‐stage hepatectomy and demonstrated the potential advantages of this technique.^62^ On an intention‐to‐treat analysis, ALPPS was successful in 92% of cases compared to 80% for classical two‐stage resection; several patients in the conventional two‐stage hepatectomy group were rescued by ALPPS.

When evaluating the effectiveness of various approaches for augmenting the future liver remnant and different types of multistage surgical procedures, no randomized controlled trial directly compares all modalities. A 2022 review aimed to assess the efficacy and safety of diverse treatment methods and make comparisons between them.^63^ Regarding liver regeneration rate, ALPPS demonstrated superiority, followed by liver venous deprivation (LVD) and portal vein embolization or ligation, aligning with existing evidence on this subject.^56^, ^64^ ALPPS also exhibited the shortest time to hepatectomy, whereas there were no significant differences between PVE, LVD, two‐stage hepatectomy, and portal vein ligation.^63^ Although ALPPS had the highest resection rate, this was not significant, compared to LVD. Additionally, no significant differences were found among the various treatment groups in terms of R0 resection rates. When examining safety, ALPPS showed a tendency towards a higher Clavien–Dindo Score ≥ 3a compared to other treatment groups, although this difference did not reach statistical significance.

In conclusion, more high‐level evidence is necessary to comprehensively weigh the pros and cons of each treatment method.

## 8 ABLATION

Ablation, primarily microwave ablation (MWA), which has superseded radiofrequency ablation (RFA), may be appropriate where surgical resection is not possible owing to technical or patient‐related factors, or in combination with a surgical resection.^65^

The CLOCC study suggested a potential benefit for ablation in addition to chemotherapy despite the small sample size. The CLOCC study was a randomized Phase 2 study in which patients with unresectable colorectal liver metastases were randomized in two groups: RFA with systemic treatment or systemic treatment alone. The median overall survival was >45 mo in the RFA with systemic treatment group versus >40 mo in the systemic only treatment group (ns). Progression‐free survival was significantly higher in the combined treatment group compared to systemic treatment only.^66^ In the follow‐up that was published in 2017, 65% of the patients in the combined and 90% of the patients in the systemic treatment had died after a median follow‐up of 9.7 y. Assessing the overall survival, there was a significantly better overall survival in the combined treatment arm. This randomized trial clearly highlights the effects of aggressive liver treatment and its benefits translating into higher survivals.^67^

The MAVERRIC trial showed similar 3‐y overall survival rates for resection and ablation for small CRLM (<3 cm).^68^ Stereotactic body radiation therapy (SBRT), also known as stereotactic ablative radiotherapy (SABR), uses precisely targeted high‐dose radiotherapy to treat small tumors, including CRLM, particularly for frail patients who may not tolerate a general anesthetic. There are no randomized studies or large studies assessing the effects of resection versus ablation versus SBRT. There is limited evidence showing good local disease control and survival in unresectable patients with SBRT.^69^ A meta‐analysis showed 2‐y survival rates of >50%.^70^

In a retrospective analysis, patients with a first recurrence of CRLM were analyzed according to their consecutive treatment method (surgery, RFA, or SBRT). The authors showed that surgery had the best local disease control.^71^

Thus, so far, the treatment of choice is still surgery, if feasible. A meta‐analysis on this topic showed an improved local control and overall survival for patients undergoing surgery for CRLM, but better evidence is pending to clearly define the role of each treatment modality.^72^

## 9 RESECTION MARGINS

Over the last two decades, there have been a handful of observational studies that have argued for and against the 1‐cm rule of CRLM cancer‐free resection margin as the minimum for curative resection.^73^, ^74^, ^75^ However, it is not always possible to achieve a wide margin, as a tumor may be in the vicinity of major vascular structures or the FLR may be small. A 1‐mm microscopic cancer‐free resection margin has been suggested by us and others to be sufficient for a curative resection. This view has now been supported by other studies, yet the debate continues.^76^, ^77^, ^78^, ^79^ The reality, however, is that to work by a 1‐cm rule will deprive many of the potential benefits of successful liver resection for CRLM.^80^

The concept of R0 versus R1 resection margins have been taken a step further forward by Torzilli and colleagues. Although accepting that R0 resection is the standard for CRLM, they debated the adequacy of R1 as a definition. Detachment of CRLM from vessels has been proposed to prioritize parenchyma‐sparing and increase resectability, and their studies have aimed to clarify the outcomes of R1 surgery (margin <1 mm), distinguishing standard R1 resection (parenchymal margin, R1Par) and R1 resection with detachment of CRLMs from major intrahepatic vessels (R1Vasc). A large study from this group included 627 resection areas in 226 consecutive patients. Fifty‐one (8.1%) resections in 46 (20.4%) patients were R1Vasc, and 177 (28.2%) resections in 107 (47.3%) patients were R1Par. Thirty‐two (5.1%) surgical margin recurrences occurred in 28 (12.4%) patients. Local recurrence risk was similar between the R0 and R1Vasc groups (per‐patient analysis 5.3% vs 4.3%; per‐resection area analysis 1.5% vs 3.9%, *P =* n.s.), but increased in the R1Par group (19.6% and 13.6%, *P* < 0.05 for both). The R1Par group had a higher rate of hepatic‐only recurrences (49.5% vs 36.1%, *P =* 0.042). On multivariate analysis, R1Par was an independent negative prognostic factor of overall survival (*P =* 0.034, median follow‐up 33 mo); conversely, R1Vasc versus R0 had no significant differences.^81^ Thus, complete tumor excision with parenchymal clearance appears to be associated with the best results: R1Par resection is not adequate for CRLM but R1Vasc surgery achieves outcomes equivalent to R0 resection. This group suggested that detachment from intrahepatic vessels can be pursued to increase patient resectability and resection safety (parenchymal‐sparing). The reason for these results may be due to the peritumoral environment. In the state of R1Par, there might already be micrometastases, whereas in the R1Vasc group the vessel itself may serve as a natural border for preventing tumor spread.^81^ Long‐term results of R1Vasc to confirm these promising results are pending.

## 10 SYNCHRONOUS DISEASE

Liver metastases discovered at the timepoint of primary colorectal cancer diagnosis are classified as synchronous disease, and this includes patients with the incidental intraoperative finding of liver metastasis. Regarding metachronous liver metastasis there has been a change in nomenclature. Patients with the finding of liver metastases up to 12 mo postdiagnosis are classified as early metachronous disease and post‐12 mo following diagnosis as late metachronous disease.^82^

Synchronous CRLM have long been assumed to represent a more aggressive spectrum of disease but there is no firm evidence to support this. Although it remains usual to deal with the primary tumor first, several studies have demonstrated equivalent survival rates and reduced hospital stays for patients undergoing synchronous resection of both primary and CRLM compared to staged resection.^83^, ^84^, ^85^, ^86^ It is clear, once again, that patient selection is paramount. Combining the first stage of two‐stage liver surgery with resection of the primary tumor is clearly another option.

In 2008, Mentha et al suggested that a liver resection‐first approach was appropriate for patients with borderline resectable CRLM who may progress while recovering from colorectal surgery, particularly if a rectal cancer had been controlled by chemoradiation.^87^ This has now been validated and, in recent years, the concept has been extended to included colonic tumors, after initial control by chemotherapy. A recent analysis of 7360 patients from the LiverMetSurvey Registry compared 552 liver‐first versus 4415 primary‐first and 2393 synchronous resections: the results were similar for all groups if the CRLM were solitary or multiple unilobar, but the liver‐first approach appeared to be superior in patients with multiple bilobar CRLM.^88^ Although perhaps counterintuitive, it is clear that in patients with synchronous CRLM, the surgical strategy should be decided according to the tumor burden. The majority of reported patients with synchronous CRLM have received neoadjuvant chemotherapy based on “expert opinion,” but this has not been based on high‐level evidence. Liver‐first approaches should be avoided in patients with locally advanced or surgically inoperable primary tumors.

## 11 OLIGOMETASTATIC DISEASE

The concept of oligometastatic disease has been popularized in recent years and may no longer be a contraindication to liver resection for CRLM. Oligometastatic disease has been described as an intermediate clinical state between localized cancer and systemically metastasized disease. Recent clinical studies have shown prolonged survival when aggressive locoregional approaches are added to systemic therapies in patients with oligometastases.^37^

Thus, resection of limited lung metastatic disease is often considered. In the presence of colorectal metastases in combination with lung metastases on imaging systemic treatment with reevaluation and rediscussion at the MDT should be the treatment option of choice, but consideration of resection and/or ablation can be a valid option.^8^, ^30^, ^89^ The case should be discussed at the thoracic MDT ideally before the primary or liver metastases are addressed.

Other sites of oligometastatic disease are, eg, peritoneum. The presence of peritoneal metastases is associated with poor outcomes.^90^ The management of this extent of disease was investigated in recent trials trying to answer whether surgery or the combination of surgery with an oxaliplatin‐based hyperthermic intraperitoneal chemotherapy (HIPEC) is beneficial in these situations.^91^ The authors showed that the addition of HIPEC did not translate into a survival benefit, and, in contrast, the addition of HIPEC in these cases led to a significant increase of complications Clavien–Dindo ≥3 at 60 d postsurgery.^91^ The authors emphasized the significance of cytoreductive surgery in such instances; however, they underscored that macroscopic radical resection is crucial. Additionally, they recommend that these carefully selected cases should be referred to specialized centers. To conclusively address this question, additional evidence is required.^92^ Currently, based on the existing evidence, cytoreductive surgery without HIPEC is still recommended.^93^, ^94^

Other rare sites of metastases include, eg, brain or bone. Limited evidence exists regarding these metastatic sites, making it challenging to offer definitive recommendations. It is crucial for these patients to be deliberated upon in multidisciplinary teams at tertiary centers to devise a personalized approach.

## 12 TRANSPLANTATION

Until recently, the only surgical option for CRLM has been liver resection. Although historic experience with liver transplantation for CRLM was poor, the Oslo group proposed revisiting transplantation a few years ago. An initial experience reported on 21 patients with an overall actuarial survival at 60% after 5 y, but it was notable that only 35% were disease‐free at the end of the first year and none were disease‐free at 2 y.^95^ However, the longer‐term survival was later confirmed at 56% after 5 y compared to only 9% with chemotherapy alone.^96^

More recent trials evaluating liver transplantation for highly selected nonresectable CRLM have shown good outcomes in well‐selected patients, and this has sparked an exponential increase in the number of patients transplanted for this indication worldwide. When compared with treatment approaches in surgical oncology, transplant oncology is faced with two unique challenges: the scarcity of organs mandates a particularly stringent selection process, and a wider breadth of expertise spanning multiple disciplines is required to produce optimal outcomes. Many countries, including the UK, have entered into service evaluation studies, as it is important to judge transplant outcomes against other accepted indications.

Another approach may be to use a small portion of a donor liver so that the rest can be used for another deserving patient; the RAPID‐concept is an example of the innovative approaches being used to drive such initiatives.^97^, ^98^, ^99^ The principle of the RAPID procedure is to transplant only a small donor graft (liver segments 2 and 3). In the subsequent phase of this procedure, once the auxiliary graft has attained adequate size and functionality, the remaining liver is excised. This procedure has reduced the ethical challenges of liver graft allocation because the remaining extended right liver graft can be given to another adult recipient.

The RAPID concept (ie, resection and partial liver segment 2–3 transplantation with delayed total hepatectomy) is still an experimental surgical procedure and should be reserved for prospective clinical trials. In the original description of the surgical technique, a resection of segments 1–3 in the recipient was advised and the venous anastomosis performed to the left hepatic vein. Since then, there have been concerns about venous outflow with graft hypertrophy, so the current recommendation is to extend the resection to segment 4 to allow the combined left and middle hepatic vein orifice to be used for venous anastomosis. Portal vein pressure is monitored after revascularization for 5 min during basal, stable conditions and during clamping of the right portal vein branch to the native liver remnant. If the pressure remains stable below 20 mm Hg, the portal vein to the right remnant liver is ligated. If the pressure is higher than 20 mm Hg during clamping, the splenic artery is ligated. If this does not alleviate graft portal hypertension, a banding of the portal vein to the right liver remnant is performed to form a stenosis that results in a stable portal pressure value to the graft of less than 20 mm Hg. If this does not reduce the portal pressure sufficiently, a portocaval shunt may be constructed using the right portal vein in an end‐to‐side fashion to the cava. CT scans with volumetry of the transplanted segment is performed at postoperative days 1, 7, 14, 21, and 28, and thereafter monthly. During the phase of graft regeneration, the portal occluded liver remnant is left in situ with immunosuppression and no chemotherapy is given in order not to negatively impact liver regeneration. As soon as the donor graft has obtained a size approaching 0.8% of body weight or 35%–40% of recipient standard liver volume, a secondary completing hepatectomy is performed, leaving only the donor segments in place. Immunosuppression is needed in the long term, as for any liver transplant.

Recently, live donor grafts have been used for the RAPID procedure with success, justified by the lower risk of donor morbidity while the liver transplant community finds a way with the ethics of transplantation for CRLM. After all, these are cases with a high disease burden and an overall poor prognosis.

## 13 CONCLUSION

In conclusion, liver resection remains the most appropriate treatment strategy for CRLM, although ablation and transplantation are developing more of a role in combination with chemotherapy. Complex cases should be referred to tertiary care centers and should be discussed formally within specialist liver cancer MDTs to clearly define therapeutic strategies for individual patients. As most treatments continue to be provided based on “expert opinion,” trials and registries are needed to further elaborate clear guidelines.

## 14 FUNDING INFORMATION

There is no funding to declare.

## CONFLICT OF INTEREST STATEMENT

Professor Lodge is an editorial board member for the *Annals of Gastroenterological Surgery*. The authors declare no conflicts of interest for this article.

## ETHICS STATEMENTS

Approval of the research protocol: N/A.

Informed Consent: N/A.

Registry and the Registration No. of the study/trial: N/A.

Animal Studies: N/A.
